# Is the Potable Water System an Advantageous Preinfection Niche for Bacteria Colonizing the Cystic Fibrosis Lung?

**DOI:** 10.1128/mBio.00883-19

**Published:** 2019-06-04

**Authors:** Matthew J. Wargo

**Affiliations:** a Department of Microbiology and Molecular Genetics, Larner College of Medicine, University of Vermont, Burlington, Vermont, USA; Georgia Institute of Technology School of Biological Sciences

**Keywords:** biofilm, cystic fibrosis, drinking water microbiology, *Pseudomonas*

## Abstract

People with cystic fibrosis are susceptible to lung infections from a variety of bacteria, a number of which also reside in the potable water system, including Pseudomonas aeruginosa, Stenotrophomonas maltophilia, Achromobacter xylosoxidans, Burkholderia cepacia complex, and nontuberculosis *Mycobacteria*. Here, I propose chemical and physical aspects of the potable water system along with bacterial lifestyle strategies in this system that may enhance successful colonization of cystic fibrosis lungs by these bacteria, including iron and copper levels, lipids, and low growth rates within low-oxygen biofilms.

## OPINION/HYPOTHESIS

Many of the bacterial pathogens that colonize lungs of people with cystic fibrosis (CF) are also residents of the potable water systems, particularly terminal plumbing and drains, including Pseudomonas aeruginosa, Stenotrophomonas maltophilia, Burkholderia cepacia complex, Achromobacter xylosoxidans, nontuberculosis *Mycobacteria*, and rarer colonizers such as *Ralstonia* spp., *Chryseobacterium* spp., and *Sphingomonas* spp. (1). There are some reports that link specific potable water strains of P. aeruginosa with infection in CF and some that rule such a link out (2–7), but there are cases where a potable water strain is a high-probability source for the infection (4, 7). Long-term (30-day) preexposure of P. aeruginosa to a model tap water mimic, Fraquil, has been shown to increase *in vitro* virulence, as measured by cytotoxicity, in a cell culture experimental model of acute infection (8), but such an analysis has not been done in the context of CF and not, to my knowledge, for any of the other CF pathogens.

The potable water system is a risk factor for a number of infections, though the overall risk for these infections is very low in countries with well-developed municipal water systems. The contribution of potable water to infection risk has been well reviewed, and the majority of infections are gastrointestinal (9). Of the potential lung pathogens, P. aeruginosa is probably the best studied of the bacteria residing in both tap water and the CF lung. Potable water and potable water-supplied recreational water (hot tubs, pools, etc.) are responsible for the bulk of P. aeruginosa folliculitis and ear infections (>80%) (10) and somewhat fewer of the cases of keratitis and intensive care unit (ICU)-acquired pneumonia (8 to 50%) (for a few examples among an extensive literature, see references 11 to 13). An important distinction is that these infections all develop acutely, and sampling of potential source environments often takes place within a few days if not during same-day surveillance. For CF, however, the time between pathogen acquisition from the source environment and detection by culture or nucleic acid-based assays is not well established. Combine this with the fact that almost nothing is known about strain diversity and temporal dynamics of these bacteria in the water system, and this issue represents a key gap in our ability to link infections to specific source environments.

The conditions under which pathogens are grown often strongly impact their virulence. For bacterial pathogens, this has been studied best for Vibrio cholerae and Legionella pneumophila. In V. cholerae, growth on phytoplankton stimulates transcription of genes important for virulence, promotes the biofilm lifestyle, and aids in survival through the gut (14, 15). For *Legionella*, ingestion by an amoeba in a potable water or ventilation system results in the most successful infection route, because the bacteria have adapted to being phagocytosed and are delivered directly to their target phagocytic cells (16). These are two well-understood paradigms, and there are many others for which some environmental impact is appreciated. This paradigm of the preinfection niche has not been as broadly applied to the (generally) extracellular bacterial pathogens residing in the tap water.

The overlap between the organisms that can infect the CF lung and the bacterial residents of the potable water system has not been lost on researchers; indeed, it has guided many of the epidemiologic studies mentioned above. It is not the general correspondence between the species that I find particularly intriguing, but rather what that correspondence might tell us about underappreciated environmental and nutritional similarities between the potable water system and the CF lung. By learning more about the biology of these bacteria during their lives in the potable water system, we may uncover important and unappreciated aspects of their pathogenesis within the CF lung. There are some specific properties that likely represent shared or similar conditions between the potable water system and the CF lung.

## IRON

Iron is an abundant dissolved metal in the potable water system as a result of both source water chemistry and the contribution of the piping material, which is iron based in many systems. Misregulation of iron homeostasis in CF results in higher iron concentrations in the airway lumen than in healthy lungs (17). The concentrations of total iron in potable water typically range from 0.01 to 5 μM (WHO guidelines) and in CF from 5 to >100 μM (18). Dealing with free iron requires appropriate adaptation to prevent oxidative damage due to Fenton’s chemistry. P. aeruginosa exposed to either tap water or CF sputum induces genes involved in iron detoxification and often reduces expression of high-affinity acquisition systems (19, 20).

## COPPER

In a manner similar to iron, there is often abundant copper in the potable water system, and the EPA regulates a safe maximum of 1.3 mg/liter (about 20 μM). Copper is not misregulated in CF (17), but phagocytes use copper-dependent oxidative stress to boost microbial killing within the phagosome (21). The concentration of copper in potable water averages 4.7 μM but ranges from undetectable to >470 μM at the tap, with high levels nearly always due to copper in the indoor plumbing fixtures (WHO guidelines). These concentrations are comparable to those in phagosomes (20 to 400 μM) (22). Exposure of bacteria to copper induces genes for copper detoxification, sequestration, and export, making these bacteria less susceptible to copper-induced killing. This has not been studied well from the pathogen preexposure side, with more focus on copper deficiencies leading to poor microbial killing on the host side (21). Preadaptation to copper stress would be predicted to enhance initial survival of bacteria transitioning to the lung from potable water and is likely just as important for non-CF infections.

## LIPIDS

Lipids, particularly those less than ∼10 carbons in length, are poorly removed by our municipal water systems, and there are small but measurable levels of lipids and phospholipid head groups within potable water (23). In the CF lung, there is abundant lipid in the sputum, derived from host lung surfactant, dying host cells, and bacteria. All of the major lipase and phospholipase systems of P. aeruginosa, some of which are important for virulence, are induced by potable water and CF sputum (19, 20). Like the benefits of preparation described for the divalent cations, production of these enzymes, particularly the hemolytic phospholipase C, PlcH, could boost bacterial survival during the transition—which was demonstrated in principle by noting greater survival from P. aeruginosa preinduced for PlcH production in a lung infection model (24).

## ADAPTATION TO LOW OXYGEN IN BIOFILMS

While the bulk of the distribution system has an appreciable oxygen content (20 to 100% O_2_ saturation), there are hypoxic regions in areas of low flow and within the biofilms that line the pipe walls in the system. Nearly all bacteria in the water system, including the opportunistic pathogens of interest here, reside in these biofilms. The mucus plugs in the CF lung have long been appreciated to have both microoxic regions and anoxia in their deepest reaches, with a steep oxygen gradient at their surface where they equilibrate with air or the tissue they contact (25–27). Being preadapted to a low-oxygen environment due to pregrowth in hypoxic biofilms might not be inherently beneficial when infecting a healthy lung, but mucus of CF patients would be an ideal landing spot for a low-oxygen-adapted cell.

## LOW GROWTH RATES IN BIOFILMS

While the nutrient environment of the CF lung is often considered relatively rich, recent work has illuminated the very low growth rates exhibited by bacterial cells in this milieu (28). These rates are not dissimilar to bacterial growth rates in tap water (29), both being on the order of double-digit hours to days. Rapidly growing cells do not transition well to environments that limit growth rate, so the slow growth of bacteria in the tap system might be an ideal preinfection behavior to integrate into a generally slow growth environment. One big difference is that while growth rates might be similar, carrying capacity is very different, with tap water (not including a surface biofilm) capable of supporting roughly 10^6^ bacteria per milliliter (29), while CF sputum can support 100 to 10,000 times more.

## SMALL ORGANIC METABOLITES

Microbial metabolism, including from anaerobes, has received quite a bit of attention in the CF microbiology community, and anaerobic metabolism has been suggested to impact the CF lung microbiota and has been linked to patient stability and exacerbation (30). From a nutritional standpoint, anaerobes produce distinct waste products after fermentative metabolism, from both carbohydrate and amino acid sources. Many of these same metabolites are among the most abundant small organic acids in potable water (23, 31), produced both by disinfection reactions and likely by anaerobic metabolism from the anoxic biofilms in the water distribution system.

## PROWLING PHAGOCYTES

As has been well studied for Legionella pneumophila, the water system is home to many types of phagocytic protozoans that, despite the evolutionary distance, work in much the same way as our phagocytes in terms of phagocytosis pathways and killing of internalized bacteria. Thus, bacteria from the potable water system are likely already primed to deal with these protozoan grazers. Preinfection response to those grazers may allow them to resist the many neutrophils and macrophages that await in the CF lung. The association with amoebae is likely particularly important for the nontuberculosis *Mycobacteria*, where association with amoeba increases virulence potential (reviewed in reference 32). Additionally, being preassociated with a surface increases P. aeruginosa killing of phagocytes (33), so that in addition to preexisting predation, growth in the potable water system as part of a biofilm could enhance survival within the CF lung.

## FINAL THOUGHTS

Here I cover some specific aspects shared between the potable water system and the CF lung. There are numerous other aspects of the potable water system that likely impact virulence or select for specific opportunistic pathogens, including fluctuating and often high temperatures (32), disinfection regimes (34, 35), and piping material choice (35). Additionally, there are almost certainly other known similarities that I have not appreciated between these two environments, as well as many that remain unknown or underappreciated. As practitioners of molecular pathogenesis, we spend much of our time thinking about bacterial life within the host. With some notable exceptions (Legionella pneumophila, Vibrio cholerae, etc.), the contribution of the niche occupied by the bacteria before infection has not been broadly appreciated for opportunistic Gram-negative bacterial pathogens. I hope that the similarities noted here become a starting point for determining the contribution of these factors to bacterial infection in CF and also a guide to identification of unknown aspects shared between these two environments.

## References

[1] Lipuma JJ 2010 The changing microbial epidemiology in cystic fibrosis. Clin Microbiol Rev 23:299–323. doi:10.1128/CMR.00068-09. 20375354 PMC2863368

[2] Barben J, Hafen G, Schmid J, Swiss Paediatric Respiratory Research Group. 2005 *Pseudomonas aeruginosa* in public swimming pools and bathroom water of patients with cystic fibrosis. J Cyst Fibros 4:227–231. doi:10.1016/j.jcf.2005.06.003. 16081326

[3] Romling U, Wingender J, Muller H, Tummler B 1994 A major *Pseudomonas aeruginosa* clone common to patients and aquatic habitats. Appl Environ Microbiol 60:1734–1738. 8031075 10.1128/aem.60.6.1734-1738.1994 PMC201555

[4] Schelstraete P, Van Daele S, De Boeck K, Proesmans M, Lebecque P, Leclercq-Foucart J, Malfroot A, Vaneechoutte M, De Baets F 2008 *Pseudomonas aeruginosa* in the home environment of newly infected cystic fibrosis patients. Eur Respir J 31:822–829. doi:10.1183/09031936.00088907. 18094014

[5] Purdy-Gibson ME, France M, Hundley TC, Eid N, Remold SK 2015 *Pseudomonas aeruginosa* in CF and non-CF homes is found predominantly in drains. J Cyst Fibros 14:341–346. doi:10.1016/j.jcf.2014.10.008. 25443472

[6] Speert DP, Campbell ME, Henry DA, Milner R, Taha F, Gravelle A, Davidson AG, Wong LT, Mahenthiralingam E 2002 Epidemiology of *Pseudomonas aeruginosa* in cystic fibrosis in British Columbia, Canada. Am J Respir Crit Care Med 166:988–993. doi:10.1164/rccm.2203011. 12359659

[7] Heirali A, McKeon S, Purighalla S, Storey DG, Rossi L, Costilhes G, Drews SJ, Rabin HR, Surette MG, Parkins MD 2016 Assessment of the microbial constituents of the home environment of individuals with cystic fibrosis (CF) and their association with lower airways infections. PLoS One 11:e0148534. doi:10.1371/journal.pone.0148534. 26859493 PMC4747485

[8] Mendis N, Lin YR, Faucher SP 2014 Comparison of virulence properties of *Pseudomonas aeruginosa* exposed to water and grown in rich broth. Can J Microbiol 60:777–781. doi:10.1139/cjm-2014-0519. 25352257

[9] Ashbolt NJ 2015 Microbial contamination of drinking water and human health from community water systems. Curr Environ Health Rep 2:95–106. doi:10.1007/s40572-014-0037-5. 25821716 PMC4372141

[10] Dziuban EJ, Liang JL, Craun GF, Hill V, Yu PA, Painter J, Moore MR, Calderon RL, Roy SL, Beach MJ, Centers for Disease Control and Prevention. 2006 Surveillance for waterborne disease and outbreaks associated with recreational water–United States, 2003–2004. MMWR Surveill Summ 55:1–30. 17183230

[11] Trautmann M, Halder S, Hoegel J, Royer H, Haller M 2008 Point-of-use water filtration reduces endemic *Pseudomonas aeruginosa* infections on a surgical intensive care unit. Am J Infect Control 36:421–429. doi:10.1016/j.ajic.2007.09.012. 18675148

[12] Bertrand X, Thouverez M, Talon D, Boillot A, Capellier G, Floriot C, Helias JP 2001 Endemicity, molecular diversity and colonisation routes of *Pseudomonas aeruginosa* in intensive care units. Intensive Care Med 27:1263–1268. doi:10.1007/s001340100979. 11511937

[13] Blanc DS, Nahimana I, Petignat C, Wenger A, Bille J, Francioli P 2004 Faucets as a reservoir of endemic *Pseudomonas aeruginosa* colonization/infections in intensive care units. Intensive Care Med 30:1964–1968. doi:10.1007/s00134-004-2389-z. 15257431

[14] Reidl J, Klose KE 2002 *Vibrio cholerae* and cholera: out of the water and into the host. FEMS Microbiol Rev 26:125–139. doi:10.1111/j.1574-6976.2002.tb00605.x. 12069878

[15] Vezzulli L, Pruzzo C, Huq A, Colwell RR 2010 Environmental reservoirs of *Vibrio cholerae* and their role in cholera. Environ Microbiol Rep 2:27–33. doi:10.1111/j.1758-2229.2009.00128.x. 23765995

[16] Lau HY, Ashbolt NJ 2009 The role of biofilms and protozoa in Legionella pathogenesis: implications for drinking water. J Appl Microbiol 107:368–378. doi:10.1111/j.1365-2672.2009.04208.x. 19302312

[17] Smith DJ, Anderson GJ, Bell SC, Reid DW 2014 Elevated metal concentrations in the CF airway correlate with cellular injury and disease severity. J Cyst Fibros 13:289–295. doi:10.1016/j.jcf.2013.12.001. 24369896

[18] Hunter RC, Asfour F, Dingemans J, Osuna BL, Samad T, Malfroot A, Cornelis P, Newman DK 2013 Ferrous iron is a significant component of bioavailable iron in cystic fibrosis airways. mBio 4:e00557-13. doi:10.1128/mBio.00557-13. PMC3753050 23963183

[19] Son MS, Matthews WJ Jr, Kang Y, Nguyen DT, Hoang TT 2007 In vivo evidence of *Pseudomonas aeruginosa* nutrient acquisition and pathogenesis in the lungs of cystic fibrosis patients. Infect Immun 75:5313–5324. doi:10.1128/IAI.01807-06. 17724070 PMC2168270

[20] English EL, Schutz KC, Willsey GG, Wargo MJ 2018 Transcriptional responses of *Pseudomonas aeruginosa* to potable water and freshwater. Appl Environ Microbiol 84:e02350-17. doi:10.1128/AEM.02350-17. 29305509 PMC5835727

[21] Hodgkinson V, Petris MJ 2012 Copper homeostasis at the host-pathogen interface. J Biol Chem 287:13549–13555. doi:10.1074/jbc.R111.316406. 22389498 PMC3340201

[22] Wagner D, Maser J, Lai B, Cai Z, Barry CE III, Honer Zu Bentrup K, Russell DG, Bermudez LE 2005 Elemental analysis of *Mycobacterium avium*-, *Mycobacterium tuberculosis*-, and *Mycobacterium smegmatis*-containing phagosomes indicates pathogen-induced microenvironments within the host cell’s endosomal system. J Immunol 174:1491–1500. doi:10.4049/jimmunol.174.3.1491. 15661908

[23] LeChevallier MW, Schulz W, Lee RG 1991 Bacterial nutrients in drinking water. Appl Environ Microbiol 57:857–862. 2039235 10.1128/aem.57.3.857-862.1991 PMC182806

[24] Ostroff RM, Wretlind B, Vasil ML 1989 Mutations in the hemolytic-phospholipase C operon result in decreased virulence of *Pseudomonas aeruginosa* PAO1 grown under phosphate-limiting conditions. Infect Immun 57:1369–1373. 2496027 10.1128/iai.57.5.1369-1373.1989 PMC313284

[25] Alvarez-Ortega C, Harwood CS 2007 Responses of *Pseudomonas aeruginosa* to low oxygen indicate that growth in the cystic fibrosis lung is by aerobic respiration. Mol Microbiol 65:153–165. doi:10.1111/j.1365-2958.2007.05772.x. 17581126 PMC4157922

[26] Cowley ES, Kopf SH, LaRiviere A, Ziebis W, Newman DK 2015 Pediatric cystic fibrosis sputum can be chemically dynamic, anoxic, and extremely reduced due to hydrogen sulfide formation. mBio 6:e00767-15. doi:10.1128/mBio.00767-15. 26220964 PMC4551978

[27] Worlitzsch D, Tarran R, Ulrich M, Schwab U, Cekici A, Meyer KC, Birrer P, Bellon G, Berger J, Weiss T, Botzenhart K, Yankaskas JR, Randell S, Boucher RC, Doring G 2002 Effects of reduced mucus oxygen concentration in airway Pseudomonas infections of cystic fibrosis patients. J Clin Invest 109:317–325. doi:10.1172/JCI13870. 11827991 PMC150856

[28] Neubauer C, Kasi AS, Grahl N, Sessions AL, Kopf SH, Kato R, Hogan DA, Newman DK 2018 Refining the application of microbial lipids as tracers of Staphylococcus aureus growth rates in cystic fibrosis sputum. J Bacteriol 200:e00365-18. doi:10.1128/JB.00365-18. 30249710 PMC6256016

[29] Vanderkooij D, Visser A, Hijnen W 1982 Determining the concentration of easily assimilable organic-carbon in drinking water. J Am Water Works Assoc 74:540–545. doi:10.1002/j.1551-8833.1982.tb05000.x.

[30] Quinn RA, Lim YW, Maughan H, Conrad D, Rohwer F, Whiteson KL 2014 Biogeochemical forces shape the composition and physiology of polymicrobial communities in the cystic fibrosis lung. mBio 5:e00956-13. doi:10.1128/mBio.00956-13. PMC3967525 24643867

[31] Westerhoff P, Mash H 2002 Dissolved organic nitrogen in drinking water supplies: a review. J Water Supply Res Technol Aqua 51:415–448. doi:10.2166/aqua.2002.0038.

[32] Claeys TA, Robinson RT 2018 The many lives of nontuberculous mycobacteria. J Bacteriol 200:e00739-17. doi:10.1128/JB.00739-17. 29483164 PMC5952384

[33] Siryaporn A, Kuchma SL, O’Toole GA, Gitai Z 2014 Surface attachment induces *Pseudomonas aeruginosa* virulence. Proc Natl Acad Sci U S A 111:16860–16865. doi:10.1073/pnas.1415712111. 25385640 PMC4250119

[34] Kotlarz N, Rockey N, Olson TM, Haig SJ, Sanford L, LiPuma JJ, Raskin L 2018 Biofilms in full-scale drinking water ozone contactors contribute viable bacteria to ozonated water. Environ Sci Technol 52:2618–2628. doi:10.1021/acs.est.7b04212. 29299927

[35] Wang H, Masters S, Hong Y, Stallings J, Falkinham JO III, Edwards MA, Pruden A 2012 Effect of disinfectant, water age, and pipe material on occurrence and persistence of Legionella, mycobacteria, *Pseudomonas aeruginosa*, and two amoebas. Environ Sci Technol 46:11566–11574. doi:10.1021/es303212a. 23046164

